# Effect of selenium on anti-Tg antibody in patients with autoimmune hypothyroidism: A randomized controlled trial

**DOI:** 10.22088/cjim.15.2.340

**Published:** 2024

**Authors:** Mina AkbariRad, Zahra Mazloum Khorasani, Behnam Beizae, Hossein Ayatollahi, Maryam Emadzadeh, Mehrdad Sarabi, Nikoo Saeedi, Negar Nekooei

**Affiliations:** 1 Department of Internal Medicine, Faculty of Medicine, Mashhad University of Medical Sciences, Mashhad, Iran; 2 Metabolic Syndrome Research Center, Mashhad University of Medical Sciences, Mashhad, Iran; 3 Department of Radiology, Faculty of Medicine, Mashhad University of Medical Sciences, Mashhad, Iran; 4 Department of Hematology and Blood Banking, Cancer Molecular Pathology Research Center, Ghaem Hospital, Mashhad University of Medical Sciences, Mashhad, Iran; 5 Clinical Research Development Unit, Ghaem Hospital, Mashhad University of Medical Sciences, Mashhad, Iran; 6 Student Research Committee, Faculty of Medicine, Mashhad University of Medical Sciences, Mashhad, Iran; 7 Student Research Committee, Islamic Azad University, Mashhad Branch, Mashhad, Iran

**Keywords:** Anti-Tg antibody, Selenium, Autoimmune hypothyroidism

## Abstract

**Background:**

The current study intends to assess the impact of oral selenium intake on anti-Tg antibody in individuals with autoimmune hypothyroidism.

**Methods:**

In this double-blinded randomized controlled trial, two groups of 72 autoimmune hypothyroid patients were randomly assigned; one group received levothyroxine (LT4) and oral selenium and the other group was given placebo with LT4. Anti-Tg antibody, free T4, anti-TPO antibody, and TSH were identified in both groups before the treatment and also 3 months after treatment and analysis of data was done by SPSS software.

**Results:**

After the intervention, the average amount of anti-Tg antibody decreased in both of the groups, and this decrease was noticeably greater in the intervention group (P = 0.03). In the intervention group, the TSH level decreased after the intervention (p < 0.05), and the free T4 level increased after the intervention (p < 0.05); the changes in these two variables were statistically significant.

**Conclusion:**

Consumption of selenium, compared to placebo, in patients with autoimmune hypothyroidism drastically reduces the level of anti-Tg antibody, and it significantly increases the free T4 level. Also, there is a greater decrease in the level of TSH compared to the control group.

Dysregulation of the immune system results in thyroid immunological injury, which causes autoimmune thyroid diseases (AITD). It is a T cell-mediated disorder (1), which is classified into Graves’s disease (GD) and Hashimoto thyroiditis (HT) (2). Although the etiology of AITD remains unknown, patients have different antibodies, the most common of which is anti-TPO. Anti-thyroglobulin antibody (anti-Tg) is the other important antibody. The immune cells and antigens can bind to this intra-follicular antibody. Massive thyroid gland destruction causes structural alterations in Tg, which trigger the creation of an anti-Tg antibody (3). Accordingly, higher levels of anti-Tg may be suggestive of worse thyroid function. Selenium (Se) is a crucial micronutrient for the body that is needed for the synthesis of selenoproteins. Many known selenoproteins, such as the enzymes which are involved in the synthesis, metabolism, and regulation of thyroid hormones, are in the thyroid gland (4-6), and of all the organs, selenium’s highest amount per gram of tissue belongs to thyroid gland (4, 7, 8). Selenocysteine is a component of all three deiodinase enzymes that convert T4 to T3, which shows the dependence of active thyroid hormone production on selenium status (7, 9). In regions where selenium deficiency is severe, thyroiditis is higher because of decreased selenium-dependent glutathione peroxidase activity in thyroid cells. On the other hand, several immune system modulators are selenium-dependent enzymes. Thus, a lack of selenium may contribute to the onset of autoimmune thyroid disorders (10), 

and higher selenium status or selenium supplementation reduces the risk of these diseases (11, 12). In some studies, the effect of selenium on reducing inflammatory activities of the thyroid and decreasing the titer of the anti-TPO antibody, anti-Tg antibody, and interleukin-2 (IL-2) is addressed (13-17).

Recent studies have suggested selenium, as an adjunctive therapy with LT4 in auto-immune thyroiditis. However, regarding to the available literature, there is no consistent clinical benefits of selenium supplementation therapy in auto-immune hypothyroidism.

 There are several clinical trials supporting the efficacy of selenium in improving both clinical presentations and laboratory findings of auto-immune hypothyroidism. For instance, it has been shown that utilizing selenium supplementation in addition to the levothyroxine therapy, resulted in decreased levels of autoimmune antibodies such as anti-Tg and anti-TPO and also decreased the serum level of TSH (18-21). In contrast with these results, Farias et al. (22) did not report any significant effect of selenium on improvement of autoimmune hypothyroid patients. Considering the lack of data to support the impact of selenium supplementation in patients with autoimmune hypothyroidism, this research attempts to establish the effect of selenium supplementation with any protective effect on thyroid autoimmunity.

## Methods

**Patients and methods: **This randomized controlled double-blinded study was conducted on newly diagnosed autoimmune hypothyroid patients from March 2017 to September 2019 at the Ghaem Hospital’s Endocrinology Department, affiliated to the Mashhad University of Medical Sciences (Mashhad, Iran). For the size of the sample, with a two-sided 5% significance level, a total of 72 participants were required. The study protocol was fully approved by the Ethics Committee of Mashhad University of Medical Sciences (IR.MUMS.fm.REC.1396.616). This research was recorded in the Iranian Registry of Clinical Trials with the number IRCT Number: IRCT20180211038689N1.

**Study Participants:** The informed consent form was signed by participants as acceptance of their participation in the study. They were 18 to 65 years old and newly diagnosed autoimmune hypothyroidism cases with a history of not resuming selenium-containing supplements for at least six months. To randomize the patients and avoid allocation bias, each patient received a sealed envelope determining their study group. Once randomly assigned, a clinician outside the research team, opened the envelopes and prescribed LT4 or LT4 plus selenium supplementation, accordingly. 

**Eligibility criteria:** Patients with a past medical history of acute systemic disease, kidney or hepatic failure, malabsorption, and heart disease were excluded from the study. In this study, the hypothyroid criteria were considered to be clinically evident hypothyroid patients with TSH greater than 5 IU/ml, and the autoimmune criteria were considered as anti-TPO antibody more than 35 IU/mL. Out of 104 patients referred to the endocrinology clinic, 20 patients had a negative anti-TPO antibody, and 12 patients did not participate in the study. Finally, the study was conducted on 72 autoimmune hypothyroid patients.

**Intervention:** All patients received levothyroxine at a dose of 50-100 g/kg (based on TSH levels) to control TSH in the normal range. Then, the patients of the intervention group received a daily dose of 200 μg selenium tablets (made by Nature Company), and the control group was given a placebo for three months. It is noteworthy that the placebo tablets were the same in appearance and packaging as the tablets of the intervention group.

**Statistical analysis:** After data gathering was completed, the data were entered and analyzed in SPSS Version 22.0 software. The mean plus standard deviation (SD), median, maximum and minimum were used to describe quantitative data, and tables were used for qualitative data. To compare the groups, independent samples t-test and chi-square tests were applied, and, if necessary, non-parametric methods like the Mann-Whitney U test were used. The sample size was calculated regarding to the standard formula for randomized controlled trials with alpha equal to 0.1 and beta to 0.2 (the test power was considered to be 80%). The total sample size was 72 patients, so 36 individuals in each group.

## Results

**Figure 1:**
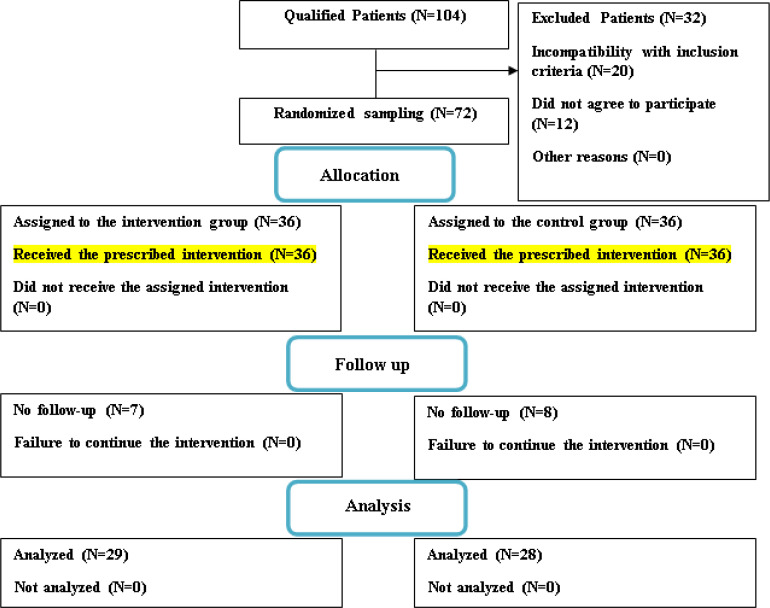
The flowchart of included patients

In this study, 104 eligible hypothyroid patients, came to the Endocrinology Clinic of Ghaem Hospital, were examined, then 32 patients were excluded of which 20 patients had a negative anti-TPO antibody and twelve patients declined to take part in the research. Eventually, out of 72 patients, 7 patients in the intervention group and 8 in the control group did not continue the follow-up (figure 1).

Table 1 displays the demographic data for the two groups, control and intervention. The patients' ages ranged from 26 to 60 years old, with a mean age of 38.40; 48 (84.2%) were females and 9 (15.8%) were males. The intervention and control groups' average ages were respectively 38.93 ± 7.47 and 37.86 ± 7.01 years, no significant difference was seen between the two groups (P-value: 0.57). In the control group, 23 (82.1%) patients were females and in the intervention group, 25 (86.2%) were females. There was no statistically significant difference in sex distribution between the two groups (P-value: 0.73). 

Table 2 shows the amount of TSH and its change in the control and intervention groups. The difference of TSH median level in the control group before and after the intervention was statistically significant. It was statistically significant as well in the intervention group before and after the intervention. Although the amount of TSH change in the intervention group was higher than the control group, the difference of changes between the two groups was not statistically significant (P-value: 0.62). 

Anti-Tg antibody’s amount is shown in table 3 and its changes in the control and intervention groups. In baseline laboratory measurements, the median serum level of anti-Tg antibody in patients was 1674.72 IU/ml, and compared with the expected laboratory range (anti-Tg antibody < 115 IU/m), 39 patients had autoimmune hypothyroidism with a high level of anti-Tg antibody. Also, 18 patients had autoimmune hypothyroidism with a high anti-TPO antibody level but a normal anti-Tg antibody level.

The anti-Tg antibody levels decreased in intervention and control groups, but the amount of decrease in the intervention group was higher. The change of anti-Tg antibody level was statistically significant in the intervention group (P=0.03). Table 4 shows the amount of free T4 and its changes in the control and intervention groups. Free T4 levels increased in both groups, however, the increase in the intervention group was greater. In addition, the change in the free T4 level was statistically significant in both groups. 

**Table 1:** Demographic and primary findings of control and intervention groups

**P-value**	**Control**	**Intervention**	**Variables**
	**Mean (± SD) or Median (Minimum-Maximum)** **or Number (%)**		
0.73^*^	23 (82.1)	25 (86.2)	**Sex (Female)**
0.57^**^	37.86 (± 7.01)	38.93 (± 7.47)	**Age (Years)**

* The two groups were compared using the Fisher test.

** To compare the two groups, independent samples t-test was used.

**Table 2:** The amount of TSH† and its changes in the control and intervention groups

**P-value**	**Control Group** **Median** **(Minimum-Maximum)**	**Intervention Group** **Median** **(Minimum-Maximum)**	**Variable**
0.89^*^	34.85 (3.95-34.39)	20 (3.5-36-98)	**TSH before intervention**
0.72^*^	6.29 (0.74-16.22)	5.29 (0.93-8.23)	**TSH after** **intervention**
0.62^*^	-7.13	-16.93	**TSH changes before and after intervention in each group**
-	P-value < 0.0001^**^	P-value < 0.0001^**^	**P-value of intra-group TSH changes after intervention**

* Mann-Whitney U test was used to compare the two groups.

** Wilcoxon test was used to compare before and after the intervention in each group.

† Thyroid stimulating hormone

**Table 3:** Anti-Tg Ab levels and its changes in the control and intervention groups

**P-value**	**Control Group** **Median (Maximum-Minimum)**	**Intervention Group** **Median (Maximum-Minimum)**	**Variable**
0.76^*^	1003.38 (4.92-1920.03)	1674.72 (6.30-2690.26)	**Anti-Tg before intervention**
0.29^*^	935.19 (2.93-1692.82)	1019.67 (1.32-2437.59)	**Anti-Tg after** **intervention**
0.84^*^	-13.60	-14.72	**Anti-Tg changes before and after intervention in each group**
-	0.09^**^	0.03^**^	**P-value of Anti-Tg changes after intervention in each group**

* Mann-Whitney test was used to compare the two groups.

** Wilcoxon test was used to compare before and after the intervention in each group.

**Table 4:** Free T4 rate and its changes in the control and intervention groups

**P-value**	**Control Group** **Mean ± SD**	**Intervention Group** **Mean ± SD**	**Variable**
0.81^*^	0.77 ± 0.28	0.79 ± 0.32	**Free T4 before intervention**
0.87^*^	1.33 ± 0.29	1.32 ± 0.39	**Free T4 after intervention**
0.75^*^	0.56 ± 0.39	0.52 ± 0.42	**Free T4 changes after intervention**
-	P-value < 0.0001^**^	P-value < 0.0001^**^	**P-value of Free T4 changes after intervention in each group**

* Independent samples test was used to compare the two groups.

** Paired samples test was used to compare before and after the intervention in each group.

## Discussion

The literature which examined the potential impact of supplemental selenium on the progress of auto-immune hypothyroidism have assessed the changes in the amount of antithyroid antibodies, and yielded an inconclusive database (23). Our outcomes showed that selenium supplementation therapy at a dose of 200 µg/day for 3 months decreased the levels of anti-Tg in serum of newly diagnosed patients with autoimmune hypothyroidism. Despite some methodological variations such as duration and dose of selenium therapy, therapeutic history of the patients, patient’s age and gender, and the severity of their disease, our findings align with those of earlier research to some extent. (18, 19, 21, 24-27). In a study by Wu et al., they aimed to investigate the clinical effects of combination therapy of selenium and levothyroxine on 18 patients with autoimmune hypothyroidism. Therapeutic effects were seen in 90% of intervention group’s patients, compared with 70% in the control group, which was a significant difference. Levels of anti-Tg antibody in the intervention group was significantly lower (67.9 vs. 89.0). Other metrics, such as T4, TSH, and anti-TPO antibody levels, were also lower in the intervention group (28). Another study by L. Yu et al. evaluated the effect of selenium on the treatment of autoimmune hypothyroidism in 60 patients. They concluded that anti-Tg antibody and TSH levels were significantly reduced, and free T4 level increased (13). In line with this, in a 2019 study, Mantovani et al. investigated the effects of selenium on the management of euthyroid patients during pregnancy, where 45 patients were evaluated at 10 and 36 weeks of gestation and 6 months after delivery (29). They reported that anti-Tg antibody levels in both groups reduced between the 10th and 36th week of gestation, but 6 months after delivery, the anti-Tg antibody levels in the placebo group increased and in the selenium group decreased. Selenium treatment did not affect T4 and TSH levels (29). 

Thus, several studies on selenium supplementation therapy in autoimmune hypothyroid patients reported no meaningful difference in anti-TG levels (16, 22). For example, Esposito et al., looked at the effect of selenium on 76 Hashimoto's euthyroid patients, they concluded that selenium had no effect on TSH, anti-TPO and anti-Tg antibody levels, but increased free T4 and reduced free T3 (20). In a meta-analysis work by Toulis et al. and based on four reviewed studies, they reported that selenium reduced anti-TPO antibody and improved patients’ quality of life and mental health, but it did not affect anti-Tg antibody, TSH and free T4 levels (16). There is still no reasonable explanation for the inconsistency among these findings. However, it might be pertained to the number of patients, duration of treatment, stage of the disease and etc.

Selenium is a key trace element which is mainly found in combination with proteins (seleno-proteins). Selenium has an essential role during synthesis, metabolism, and activation of thyroid hormones (30). The thyroid is ranked as the third selenium rich organ in the body. Glutathione peroxidase is known as a selenium containing protease that possess a strong antioxidant potential which removes excess peroxides and reactive free oxygens in thyroid. Its function results in maintenance of the cell wall and affects the synthesis process of thyroid hormones (7). The other selenoenzyme is iodothyronine deiodinase participating in metabolism of thyroid hormones. Selenium deprivation may result in impaired hormone synthesis and reduced antioxidant effects. It may also lead to destruction of thyroid glandular cells, increased Th1/Th2 ratio, and increase immune response which are important mechanisms in autoimmune hypothyroidism. Previous studies have demonstrated that selenium supplements reduced thyroid autoantibody levels, downregulated cytokines, and decreased immune response in autoimmune thyroid diseases (28). According to our study's findings, consuming selenium lowers anti-Tg antibody and TSH levels in people with autoimmune hypothyroidism and increases the level of free T4 in those patients. However, due to the current study's limitations, including the sample size and lack of selenium level measurement before and after the intervention, the evidence is insufficient to prove that selenium supplementation is effective for treating autoimmune hypothyroidism in patients. Therefore, there is a need for additional research with an expanded sample size and different doses of selenium and other factors which affect the level of anti-Tg antibodies. 

## References

[1] Antonelli A, Ferrari SM, Corrado A, Di Domenicantonio A, Fallahi P (2015). Autoimmune thyroid disorders. Autoimmun Rev.

[2] Rydzewska M, Jaromin M, Pasierowska IE, Stożek K, Bossowski A (2018). Role of the T and B lymphocytes in pathogenesis of autoimmune thyroid diseases. Thyroid Res.

[3] Barin J, Talor M, Sharma R, Rose N, Burek CL (2005). Iodination of murine thyroglobulin enhances autoimmune reactivity in the NOD. H2h4 mouse. Clin Exp Immunol.

[4] Duntas LH, Benvenga S (2015). Selenium: an element for life. Endocrine.

[5] Schomburg L (2012). Selenium, selenoproteins and the thyroid gland: interactions in health and disease. Nat Rev Endocrinol.

[6] Köhrle J (2005). Selenium and the control of thyroid hormone metabolism. Thyroid.

[7] Duntas LH (2010). Selenium and the thyroid: a close-knit connection. J Clin Endocrinol Metab.

[8] Köhrle J, Gärtner R (2009). Selenium and thyroid. Best Pract Res Clin Endocrinol Metab.

[9] Duntas LH (2006). The role of selenium in thyroid autoimmunity and cancer. Thyroid.

[10] Gärtner R, Gasnier BC, Dietrich JW, Krebs B, Angstwurm MW (2002). Selenium supplementation in patients with autoimmune thyroiditis decreases thyroid peroxidase antibodies concentrations. J Clin Endocrinol Metab.

[11] Wu Q, Rayman MP, Lv H (2015). Low population selenium status is associated with increased prevalence of thyroid disease. J Clin Endocrinol Metab.

[12] Rayman MP (2012). Selenium and human health. Lancet.

[13] Yu L, Zhou L, Xu E (2017). Levothyroxine monotherapy versus levothyroxine and selenium combination therapy in chronic lymphocytic thyroiditis. J Endocrinol Invest.

[14] van Zuuren EJ, Albusta AY, Fedorowicz Z, Carter B, Pijl H (2013). Selenium supplementation for Hashimoto's thyroiditis. Cochrane Database Syst Rev.

[15] Krysiak R, Okopien B (2011). The effect of levothyroxine and selenomethionine on lymphocyte and monocyte cytokine release in women with Hashimoto's thyroiditis. J Clin Endocrinol Metab.

[16] Toulis KA, Anastasilakis AD, Tzellos TG, Goulis DG, Kouvelas D (2010). Selenium supplementation in the treatment of Hashimoto's thyroiditis: a systematic review and a meta-analysis. Thyroid.

[17] Negro R (2008). Selenium and thyroid autoimmunity. Biologics.

[18] Wu D, Jin L, Xu H (2018). Clinical effects of selenium yeast and levothyroxine combined therapy on patients with lymphocytic thyroiditis. Biomed Res.

[19] Yu L, Zhou L, Xu E (2017). Levothyroxine monotherapy versus levothyroxine and selenium combination therapy in chronic lymphocytic thyroiditis. J Endocrinol Invest.

[20] Esposito D, Rotondi M, Accardo G (2017). Influence of short-term selenium supplementation on the natural course of Hashimoto’s thyroiditis: clinical results of a blinded placebo-controlled randomized prospective trial. J Endocrinol Invest.

[21] Mantovani G, Isidori A, Moretti C (2019). Selenium supplementation in the management of thyroid autoimmunity during pregnancy: results of the “SERENA study”, a randomized, double-blind, placebo-controlled trial. Endocrine.

[22] De Farias C, Cardoso B, De Oliveira G (2015). A randomized-controlled, double-blind study of the impact of selenium supplementation on thyroid autoimmunity and inflammation with focus on the GPx1 genotypes. J Endocrinol Invest.

[23] Schomburg L (2012). Selenium, selenoproteins and the thyroid gland: interactions in health and disease. Nat Rev Endocrinol.

[24] Gärtner R, Gasnier BC, Dietrich JW, Krebs B, Angstwurm MW (2002). Selenium supplementation in patients with autoimmune thyroiditis decreases thyroid peroxidase antibodies concentrations. J Clin Endocrinol Metab.

[25] Duntas LH, Mantzou E, Koutras DA (2003). Effects of a six month treatment with selenomethionine in patients with autoimmune thyroiditis. Eur J Endocrinol.

[26] Turker O, Kumanlioglu K, Karapolat I, Dogan I (2006). Selenium treatment in autoimmune thyroiditis: 9-month followup with variable doses. J Endocrinol.

[27] Mazokopakis EE, Papadakis JA, Papadomanolaki MG (2007). Effects of 12 months treatment with L-selenomethionine on serum anti-TPO levels in patients with Hashimoto's thyroiditis. Thyroid.

[28] Tan L, Sang Z, Shen J (2013). Selenium supplementation alleviates autoimmune thyroiditis by regulating expression of TH1/TH2 cytokines. Biomed Environ Sci.

[29] Mantovani G, Isidori A, Moretti C (2019). Selenium supplementation in the management of thyroid autoimmunity during pregnancy: results of the “SERENA study”, a randomized, double-blind, placebo-controlled trial. Endocrine.

[30] Rasmussen LB, Schomburg L, Köhrle J (2011). Selenium status, thyroid volume, and multiple nodule formation in an area with mild iodine deficiency. Eur J Endocrinol.

